# Protective Effects of Pentoxifylline on Peripheral Microcirculatory Dysfunction and Renal Cortical Oxygen Deficiency in a Rat Model of LPS‐Induced Sepsis

**DOI:** 10.1111/micc.70056

**Published:** 2026-03-09

**Authors:** Bülent Ergin, H. Rob Taal, Deniz Erol Kutucu, Wijnie van Dam, Aysegul Kapucu, Sinno S. H. P. Simons, Can Ince, Irwin K. M. Reiss

**Affiliations:** ^1^ Department of Intensive Care Adult, Laboratory of Translational Intensive Care Erasmus Medical Center, Erasmus University Rotterdam Rotterdam the Netherlands; ^2^ Department of Neonatal and Pediatric Intensive Care, Erasmus MC Sophia Children's Hospital University Hospital Rotterdam Rotterdam the Netherlands; ^3^ Department of Zoology, Faculty of Science University of Istanbul Istanbul Turkey; ^4^ Department of Neonatology and Pediatric Intensive Care Medicine University Medical Center Hamburg‐Eppendorf Hamburg Germany

**Keywords:** microcirculation, oxygenation, renal dysfunction, sepsis

## Abstract

**Objective:**

Sepsis is associated with hypotension, tissue hypoperfusion, and microcirculatory dysfunction leading to multi‐organ failure and mortality. Pentoxifylline (PTX), a phosphodiesterase inhibitor, is reported to improve blood flow and viscosity. The aim of this study was to investigate the efficacy of PTX on peripheral and renal microcirculatory alterations in sepsis.

**Methods:**

Fully instrumented Wistar albino rats were randomized as the control group (only surgery), only lipopolysaccharide (LPS) group without treatment (T1), LPS group with PTX treatment (LPS + PTX), LPS group treated with only fluid resuscitation (Ringer's acetate; RA), LPS + RA, LPS group with combined treatment with PTX and RA (LPS + PTX + RA) for 3 h (T2, T3 and T4). The systemic hemodynamics, renal oxygenation, leg muscle microcirculation, histological damage, and inflammatory and endothelial injury markers were analyzed.

**Results:**

The renal cortical microvascular oxygen pressure (cμPO_2_) was improved by the PTX, RA, and PTX + RA treatments compared to the LPS group (*p* < 0.05). The proportions of perfused vessels (PPV) and red blood cell velocity (RBCv) were significantly restored by PTX and RA compared with the LPS group at T4 (*p* < 0.05). Renal damage and inflammatory cell infiltration were reduced by PTX and RA together compared with RA alone (*p* < 0.05).

**Conclusion:**

In this study, we found that PTX may protect renal oxygenation, peripheral (muscle) microcirculation, renal damage, and tissue inflammatory cell infiltration in a rat model of LPS‐induced sepsis.

**Abbreviations**
cAMP: cyclic adenosine monophosphate
CaO_2_
: arterial oxygen content
cGMP: cyclic guanosine monophosphate
cmPO_2_
: renal cortical microvascular oxygen pressure
CRP: C‐reactive protein
CvO_2_
: venous oxygen content
DO_2ren_
: renal oxygen delivery
ELISA: enzyme‐linked immunosorbent assay
FCD: functional capillary density
FiO_2_
: fraction of inspired oxygen
GFR: glomerular filtration rate
HA: hyaluronic acid
HR: heart rate
HWM: hand‐held vital microscope
ICU: intensive care unit
IL‐6: interleukin‐6
LPS: lipopolysaccharide
MAP: mean arterial pressure
NGAL: the neutrophil‐gelatinase‐associated lipocalin
PaO_2_
: arterial partial pressure of oxygen
PCO_2_
: carbon dioxide partial pressure
PEEP: pulmonary end‐expiratory pressure
Pins: inspiratory pressure
PPV: proportion of perfused vessel
PrvO_2_
: renal vein partial oxygen pressure
PTX: pentoxifylline
RA: Ringer's Acetate
RBCv: red blood cell velocity
RBF: renal blood flow
rcBF: renal cortical blood flow
rstO_2_
: renal capillary hemoglobin oxygen saturation
SaO_2_
: arterial hemoglobin oxygen saturation
SrvO_2_
: renal vein hemoglobin oxygen saturation
TNF‐α: tumor necrosis factor‐alpha
tRBCp: tissue red blood perfusion
Tv: tidal volume
VO_2ren_
: renal oxygen consumption

## 1 Introduction

Sepsis is described as a life‐threatening condition that is associated with hypotension, tissue hypoperfusion, microcirculatory dysfunction, and dysregulated immune response to infection [1]. Despite many advanced diagnostic and therapeutic tools, sepsis remains one of the significant reasons for multiple organ failure and death among patients in intensive care units (ICU). In addition to inflammation, microcirculatory dysfunction is considered a key therapeutic target for reducing sepsis‐induced multiple organ dysfunction and mortality.

Microcirculatory effects of sepsis include tissue hypoperfusion, cell hypoxia, endothelial barrier dysfunction, inadequate oxygen utilization, loss of vascular tone, and cell death [2, 3]. Until now, many vasoactive, antioxidant, and anti‐inflammatory drugs have been successfully used to mitigate the microcirculatory alterations of sepsis, both clinically and experimentally [4, 5, 6]. However, more clinically available, valid, and reliable compounds/drugs are needed to investigate the potential for improving microcirculatory perfusion and oxygenation in both peripheral tissues and organs. Pentoxifylline (PTX) is a non‐selective phosphodiesterase inhibitor commonly used to treat late‐onset neonatal sepsis and necrotizing enterocolitis in neonates, in addition to antibiotic therapy [7]. However, the systematic meta‐analysis revealed that the positive effect of PTX on mortality and hospital stay is based on low‐quality evidence [7]. In the microcirculation, PTX is reported to improve blood flow and red blood cell deformability, reduce blood viscosity, and reduce platelet aggregation and thrombus in intermittent claudication [8]. However, there is a lack of evidence regarding the effects of PTX on sepsis‐induced peripheral and organ‐based microcirculation, including the susceptible kidneys.

Therefore, the aim of this study was to investigate the efficacy of PTX on peripheral and renal microcirculatory alterations in a rat model of LPS‐induced sepsis, to give more insight into the consequences of using PTX on macro‐ and microcirculation, and to underline the importance of microcirculatory targeted therapy in ICU settings.

## 2 Material and Methods

### 2.1 Animals

This study was approved by the National Committee of Animal Experimentation (CCD172326, approval date: 05‐03‐2018) and the Animal Research Committee of the Erasmus Medical Centre, Rotterdam (SP2100292). Care and handling of the animals were performed in accordance with the Institutional Animal Care and Use Committees and the ARRIVE guidelines. Thirty‐five male Wistar albino rats (Mean ± SD bodyweight of 357 ± 29 g) were used in 5 groups (Charles River, The Netherlands) with seven animals per group.

### 2.2 Surgical Preparation

All rats were sedated with an intraperitoneal injection of a mixture of 90 mg/kg ketamine (Nimatek, Eurovet, Bladel, The Netherlands), 0.5 mg/kg dexmedetomidine (Dexdomitor, Pfizer Animal Health BV, Capelle aan den IJssel, The Netherlands), and 0.05 mg/kg atropine‐sulfate (Centrafarm Pharmaceuticals BV, Etten‐Leur, The Netherlands). Maintenance of anesthesia was achieved by 50 mg·kg^−1^·h^−1^ Ketamine and 12.5 μg·kg^−^1·h^−1^ dexmedetomidine in 5 mL·kg^−1^·h^−1^ Ringer's Acetate solution (Sterofundin, BBraun, Germany) (RA). Fluid balance was maintained with 15 mL·kg^−1^·h^−1^ continuous infusion of RA. Following tracheotomy, ventilator settings were adjusted as Tidal volume (Tv): 8% of Body Weight (BW), fraction of inspired oxygen (FiO_2_): 0.4, pulmonary end expiratory pressure (PEEP): 3 cmH_2_O, and inspiratory pressure (Pinsp): 15–20 cmH_2_O. The body temperature was maintained at 37°C ± 0.5°C by a heating pad under the animal. The end‐tidal PCO_2_ was kept between 40 and 45 mmHg, corresponding to 30–35 mmHg in the blood.

The right carotid artery was cannulated with a polyethylene catheter (outer diameter = 0.9 mm, Braun, Melsungen, Germany) primed with heparinized saline to measure arterial blood pressure (MAP) and heart rate (HR) using a pressure transducer (ADInstruments Limited, Oxford, UK). The catheter inserted into the right jugular vein was used for fluid support and maintenance of anesthesia during the experiment. The catheter in the left femoral artery was used for blood sampling, and the one in the left femoral vein (for LPS and drug infusion) was allocated for LPS, pd‐porphyrin, and PTX infusion. The left kidney was exposed and stabilized in a kidney cup via a 3 cm retroperitoneal incision. The oxygen quenching of pd‐porphyrin phosphorescence methods was used to continuously measure renal cortical partial oxygen pressure (cμPO_2_) [9, 10]. An ultrasonic flow probe was placed around the left renal artery (type 0.7 RB; Transonic Systems Inc., Ithaca, NY, USA) to assess renal blood flow (RBF) throughout the experiment. The biceps femoris of the left hindlimb was used for measuring microcirculation with a handheld vital microscope (HVM; CytoCam, Braedius, The Netherlands). To prevent muscle desiccation, the exposed area was protected with a humidified gauze with warm 0.9% NaCl. The animals rested for 30 min after completion of surgery (30 min).

### 2.3 Experimental Protocol

Rats were randomized into five groups, with at least *n* = 7 per group (a total of 35 rats), according to the power analysis (G*power, HHU, Germany). Endotoxemia was induced by 10 mg^.^kg^−1^ gram‐negative bacterial lipopolysaccharide infusion (LPS; serotype 0127:B8) (Sigma‐Aldrich Corporation, St Louis, MO, USA) over 30 min (dissolved in 1 mL of 0.9% NaCl solution) [11], followed by a cessation period (~3 h) till MAP reaches below 65 mmHg (T1) (Shock).

Following the induction of sepsis (MAP < 65 mmHg) (T1), we randomized the experimental groups accordingly: Control group (only surgery), the LPS group received 10 mg^.^kg^−1^ LPS without treatment (LPS), the LPS group with PTX treatment (Trental, Sanofi BV, Amsterdam, The Netherlands) for 3 h at the dose of 60 mg^.^kg^−1^ [12] (LPS + PTX), only fluid resuscitation (30 mL·kg^−1^ h^−1^, RA) [13] (LPS + RA) and combination treatment group with both PTX and RA group (LPS + PTX + RA) for 3 h of observation (T2, T3 and T4). 0.1 mL of arterial blood was used to determine blood gas, electrolytes, and hemoglobin (RP 500 Blood gas analyzer, Siemens Health, The Netherlands).

### 2.4 Renal Oxygenation

An optical fiber with a tip diameter of 5 mm was placed ~1 mm above the exposed kidney to measure oxygenation using a phosphorescence lifetime technique described elsewhere [10]. Palladium (II)‐meso‐tetra (4‐carboxyphenyl)‐porphyrin (Pd‐TCCP) at a concentration of ~5 mg·mL^−1^ was injected at a dose of 1 mL·kg^−1^ and excited at 530 nm. Analysis of the phosphorescent decay time allows quantitative measurement of the microcirculatory cμPO_2_ in the kidney, and it was continuously recorded in LabView 2014 (National Instruments, Austin, TX, USA) as described previously. The renal capillary oxygen saturation (rstO_2_) and renal cortical blood flow (rcBF) were measured by the O2C system (LEA Medical, Germany).

### 2.5 Renal Oxygen Delivery and Consumption

Arterial oxygen content (CaO_2_) was calculated by the following equation: (1.31 × hemoglobin × SaO_2_) + (0.003 × PaO_2_), where SaO_2_ is arterial oxygen saturation, and PaO_2_ is the arterial partial pressure of oxygen. Renal delivery of O_2_ (DO_2ren_) expressed as mL O_2_·min^−1^ was determined at the end of the experiment based on the equation: DO_2ren_ = (CaO_2_) × RBF. Blood gas analysis was performed on 0.5 mL samples of renal venous blood (obtained by venopuncture at the end of the experiment). Venous oxygen content (CvO_2_) was determined as (1.31 × hemoglobin × SrvO_2_) + (0.003 × PrvO_2_), where SrvO_2_ is venous oxygen saturation, and PrvO_2_ is renal vein partial pressure of oxygen. Renal oxygen consumption (VO_2ren_) (mL O_2_.min^−1^) was determined based on the following equation: VO_2ren_ = C (arterial–venous)O_2_ × RBF.

### 2.6 Muscle Microcirculation and Oxygen Saturation

A hand‐held vital microscope (HVM), CytoCam (Braedius Scientific, Huizen, The Netherlands), was placed on the surface of the exposed biceps femoris with the help of a micromanipulator [4]. A one‐hundred‐frame clip (100 s) was recorded at each time point. The microcirculatory parameters, such as functional capillary density (FCD), proportion of perfused vessels (PPV), red blood cell velocity (RBCv), and tissue red blood perfusion (tRBCp) were analyzed by Microtools automated software [14].

### 2.7 Inflammation and Histological Assessment

Interleukin‐6 (IL‐6), and tumor necrosis factor‐alpha (TNF‐α), C reactive protein (CRP) (R&D System Inc., Minneapolis, USA), and hyaluronic acid levels were assessed by enzyme‐linked immunosorbent assay (ELISA) in plasma. The neutrophil‐gelatinase‐associated lipocalin (NGAL) (R&D System Inc., Minneapolis, USA) was also evaluated for renal damage.

Kidney tissues were fixed in 4% formalin and embedded in paraffin. Kidney sections (4 μm) were deparaffinized with xylene, rehydrated with stepwise decreasing ethanol concentrations, and finally water. The kidney sections were stained with periodic acid‐Schiff and hematoxylin to assess histological changes in the kidney by quantitative measurements of tissue damage. The degree of kidney damage of medulla and cortex was estimated at 400× magnification using 10 randomly selected fields for each animal by the following criteria: 0, involving 1%–10% areas with damage; 1, 10%–25% areas with damage; 2, 25%–50% tubular damage; 3, 50%–75% area of damage; and 4, more than 75% areas with damage. The degree of kidney inflammation in the medulla and cortex was evaluated at 400× magnification using 10 randomly selected fields for each animal. Inflammatory score was classified as the percentage area infiltrated by immune cells in hematoxylin–eosin stained slides: 1: none, 2: less than 25% area, 3: 25%–50% area, 4: 50%–75% area, and 5: more than 75% area [15].

### 2.8 Statistical Analysis

Values are expressed as mean ± standard error of mean (SEM) when the data are fitted in a Gaussian distribution (Kolmogorov–Smirnov test). Repeated measures of two‐way analysis of variance (RM‐ANOVA) with the Geisser–Greenhouse correction test or mixed models with Tukey's multiple comparison test were used to determine intra‐intergroup differences in hemodynamics data. Ordinary one‐way ANOVA with Tukey's multiple comparison test was used for intergroup analysis between the experimental groups at T4. The overall significance level for each hypothesis was 0.05. Adjusted *p*‐values were reported throughout the manuscript in post hoc tests. Statistical analyses were performed using GraphPad Prism 9 (San Diego, CA, USA).

## 3 Results

### 3.1 Systemic Hemodynamics

In this septic model, MAP decreased significantly after the induction of sepsis (LPS vs. Control BL at T1, T2, T3, and T4, *p* < 0.05), and none of the used therapies restored it after 3 h (T2). HR is significantly increased in the LPS + PTX + RA group at T2 and T3 and the LPS + PTX group at T3, compared to the control group. The LPS + PTX at T3 and the LPS + PTX + RA group at T3 and T4 showed an increased HR compared to the LPS alone. Lastly, plasma lactate levels are increased in the LPS group at T3 and T4, in the LPS + PTX group at T3 and T4, and in the LPS + PTX + RA group at T4, compared to the Control group. At T4, lactate level stays high in LPS + PTX + RA compared to LPS + RA (*p* < 0.05) (Table 1).

**TABLE 1:** Systemic hemodynamic parameters and oxygenation.

	BL	T1	T2	T3	T4
MAP (mmH)g					
Control	99 ± 5	94 ± 4	84 ± 6	74 ± 6	69 ± 2
LPS	102 ± 5	62 ± 1*	47 ± 2*	40 ± 2*	31 ± 2*
LPS + PTX	97 ± 4	60 ± 3*	49 ± 2*	42 ± 3*	32 ± 3*
LPS + RA	97 ± 4	59 ± 1*	57 ± 2* + #	56 ± 3+	52 ± 3*+#
LPS + PTX + RA	85 ± 3	62 ± 1*	53 ± 2*	56 ± 4+	48 ± 2*+#
HR (BPM)					
Control	254 ± 7	248 ± 4	258 ± 7	280 ± 6	301 ± 10
LPS	264 ± 11	260 ± 6	265 ± 7	269 ± 8	247 ± 11*
LPS + PTX	268 ± 6	265 ± 5	280 ± 9	316 ± 7*+#	294 ± 13+
LPS + RA	248 ± 9	267 ± 11	266 ± 6	269 ± 5#	255 ± 5*
LPS + PTX + RA	265 ± 7	257 ± 6	300 ± 9*	330 ± 10* + $	328 ± 7+$
Lactate (mmol/L)					
Control	1.2 ± 0.1	2.3 ± 0.2	2.2 ± 0.2	1.5 ± 0.1	1.9 ± 0.2
LPS	1.3 ± 0.1	2.3 ± 0.1	2.2 ± 0.2	2.7 ± 0.3*	4.2 ± 0.3*
LPS + PTX	1.3 ± 0.1	2.2 ± 0.2	2.1 ± 0.1	3.4 ± 0.4*	6 ± 0.5*
LPS + RA	1.4 ± 0.1	2.6 ± 0.2	1.9 ± 0.1	2 ± 0.1	2.5 ± 0.2+#
LPS + PTX + RA	1.8 ± 0.2	2.8 ± 0.3	1.7 ± 0.1	2.6 ± 0.4	4.2 ± 0.3*$

*Note:* Values are represented as mean ± SEM. *p < 0.05 vs. Control, +*p* < 0.05 vs. LPS, #*p* < 0.05 vs. LPS + PTX, $*p* < 0.05 vs. LPS + RA.

Abbreviations: HR, heart rate; MAP, mean arterial pressure.

### 3.2 Renal Oxygenation and Perfusion

**FIGURE 1:**
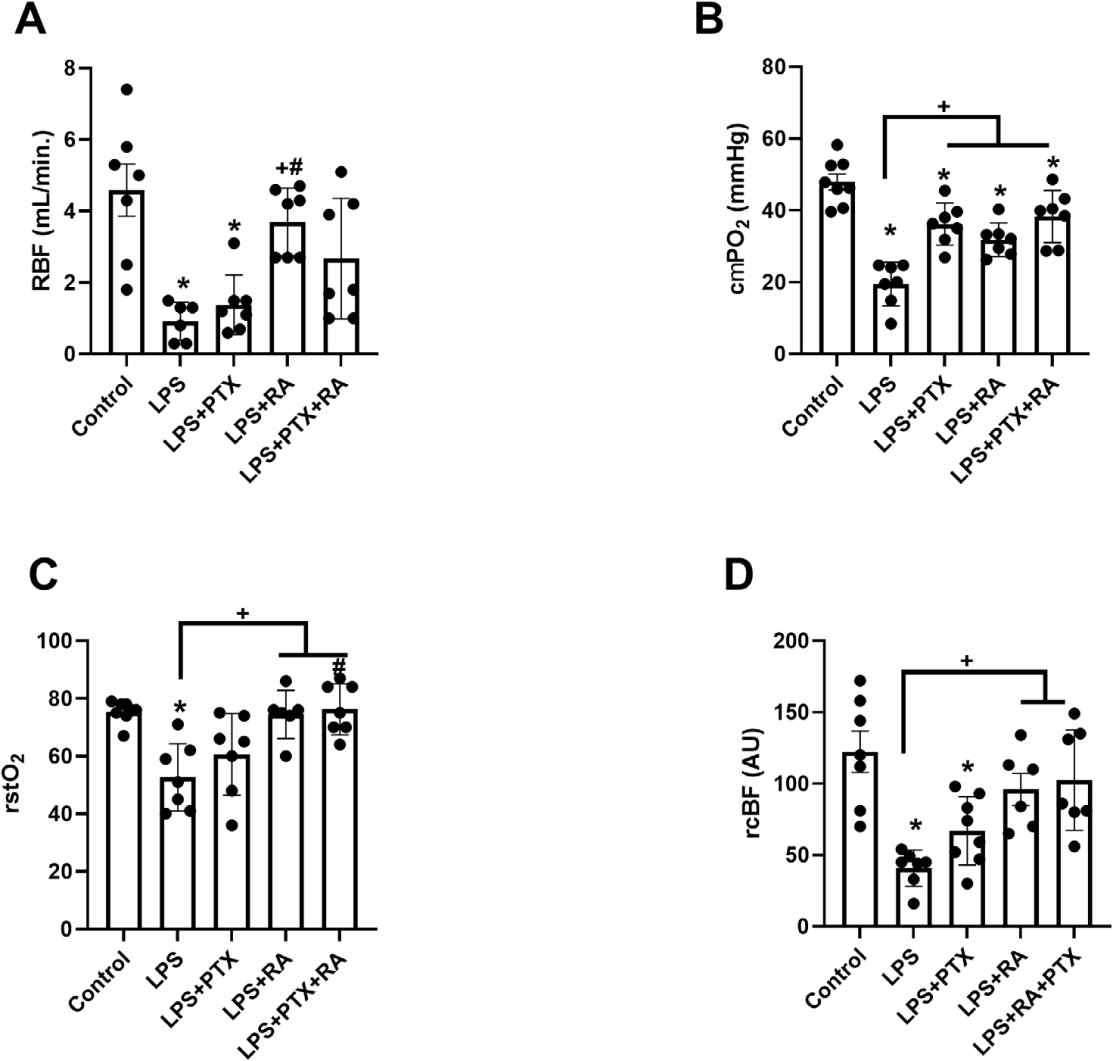
Renal cortical oxygenation and perfusion. Renal blood flow (RBF) (A), cortical partial oxygen pressure (cμPO_2_) (B), cortical oxygen saturation (rstO_2_) (C), and cortical blood flow (rcBF) (D) at the end of experiments (T4). Values are represented as mean ± SEM. *p < 0.05 vs. Control, ^+^ *p* < 0.05 vs. LPS, ^#^ *p* < 0.05 vs. LPS + PTX.

RBF was significantly reduced in the LPS group, and the LPS group received PTX at T4 compared to the control (*p* < 0.05). However, the LPS group received only RA has higher RBF than the LPS and LPS + PTX groups (*p* < 0.05) (Figure 1A).

The cμPO_2_, measured by the oxygen quenching phosphorescence technique, showed a significant reduction in the LPS group compared to the Control group (*p* < 0.05). This reduction was improved by the PTX, RA, and PTX + RA treatments compared to the LPS group (*p* < 0.05) (Figure 1B). rstO_2_ and rcBF decreased in the LPS group (*p* < 0.05 vs. Control) and were restored only in the LPS group that received fluid support (*p* < 0.05 vs. LPS) (Figure 1C,D).

**FIGURE 2:**
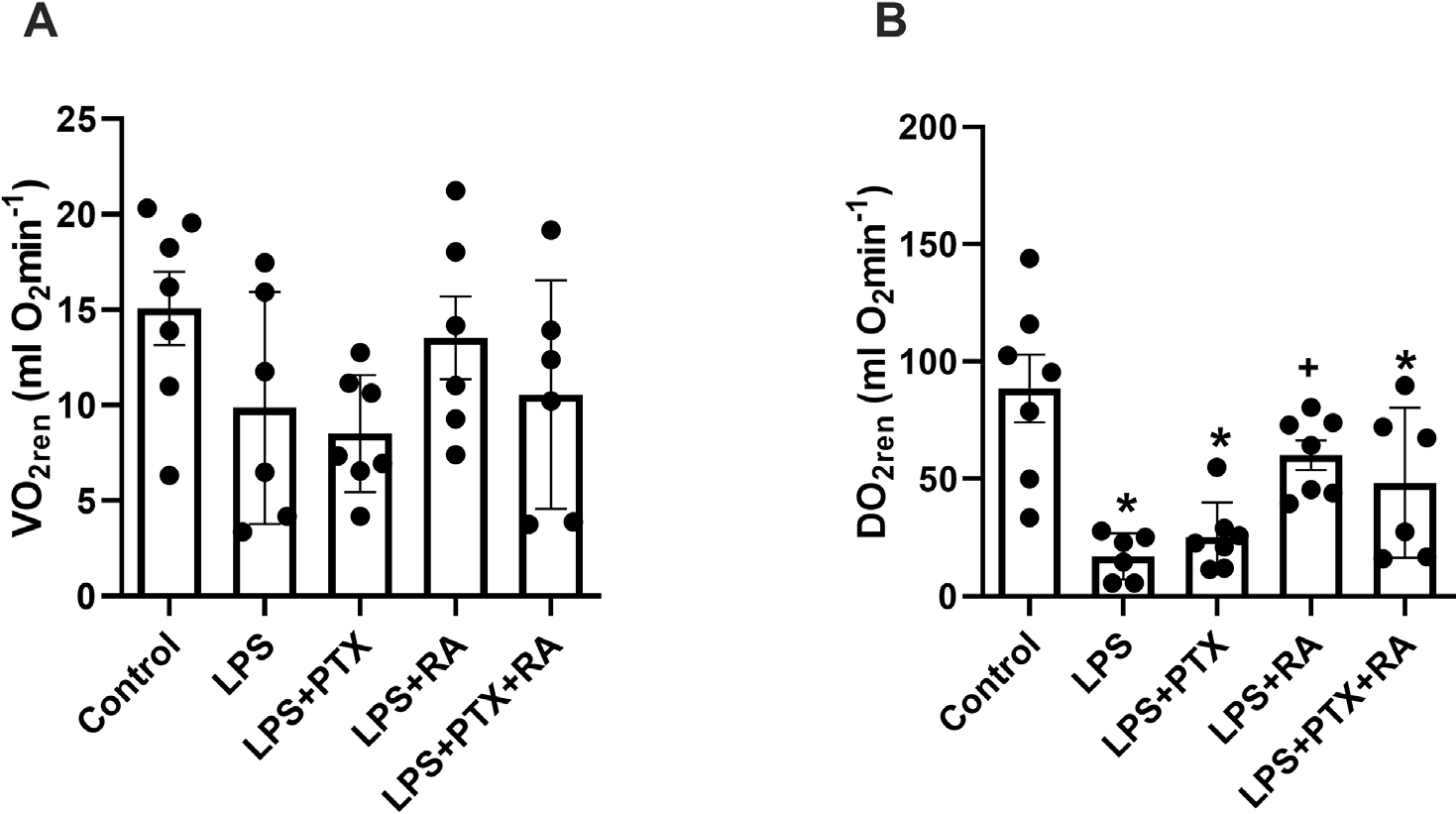
Renal oxygen consumption (VO_2ren_) (A) and delivery (DO_2ren_) (B) at the end of experiments (T4). Values are represented as mean ± SEM. *p < 0.05 vs. Control, ^+^ *p* < 0.05 vs. LPS.

No change in renal oxygen consumption was found among the groups (Figure 2A). Renal DO_2_ is reduced after LPS induction (*p* < 0.001) and remains unchanged in the LPS group that received PTX. Only the LPS + RA group showed a significant improvement in DO_2ren_ compared to the LPS group (*p* < 0.05) (Figure 2B).

### 3.3 Renal Damage and Inflammatory Cell Infiltration

**FIGURE 3:**
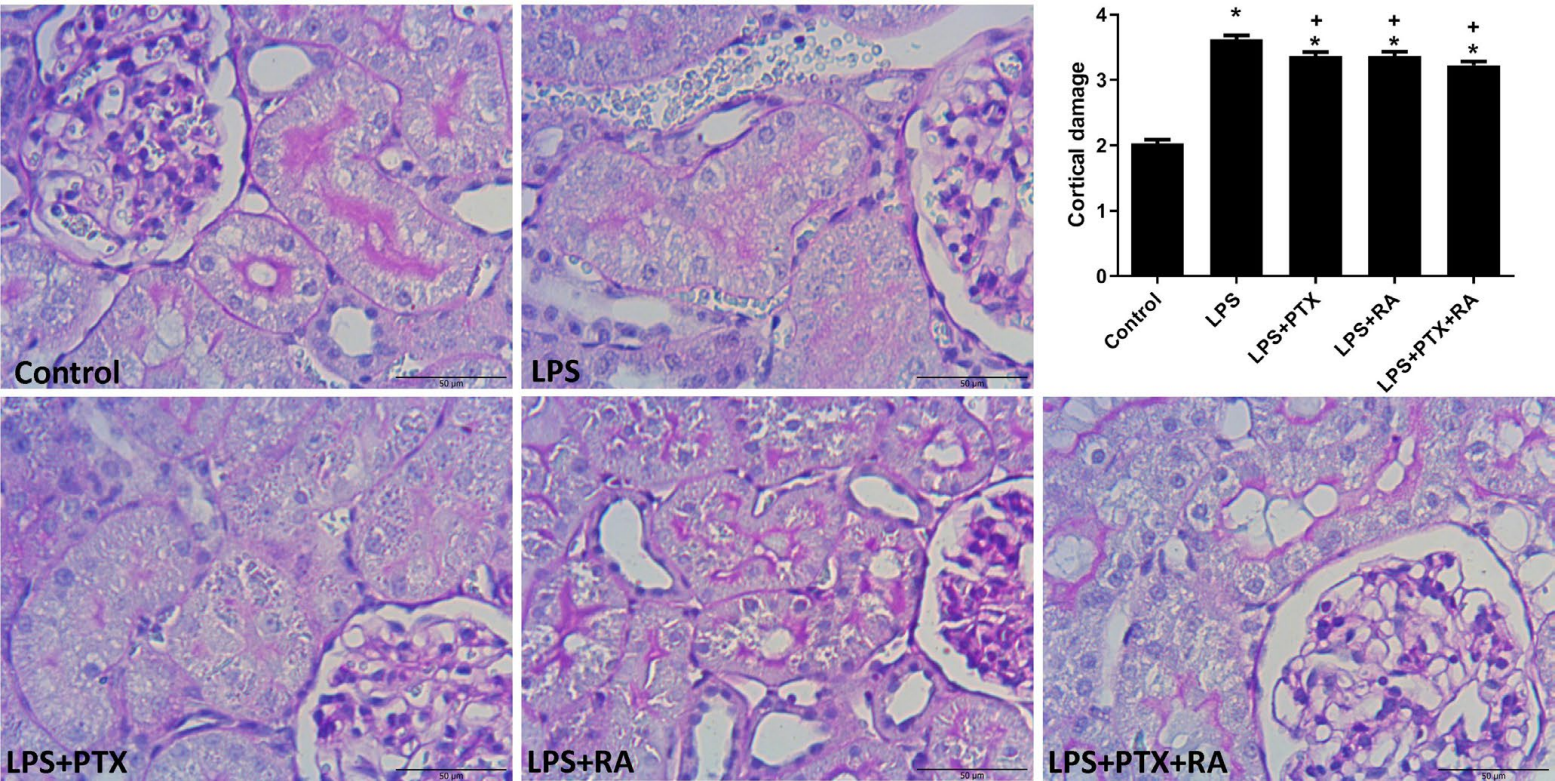
Representative images of renal cortical damage and damage score among the experimental groups. Values are represented as mean ± SEM. *p < 0.05 vs. Control, ^+^ *p* < 0.05 vs. LPS.

**FIGURE 4:**
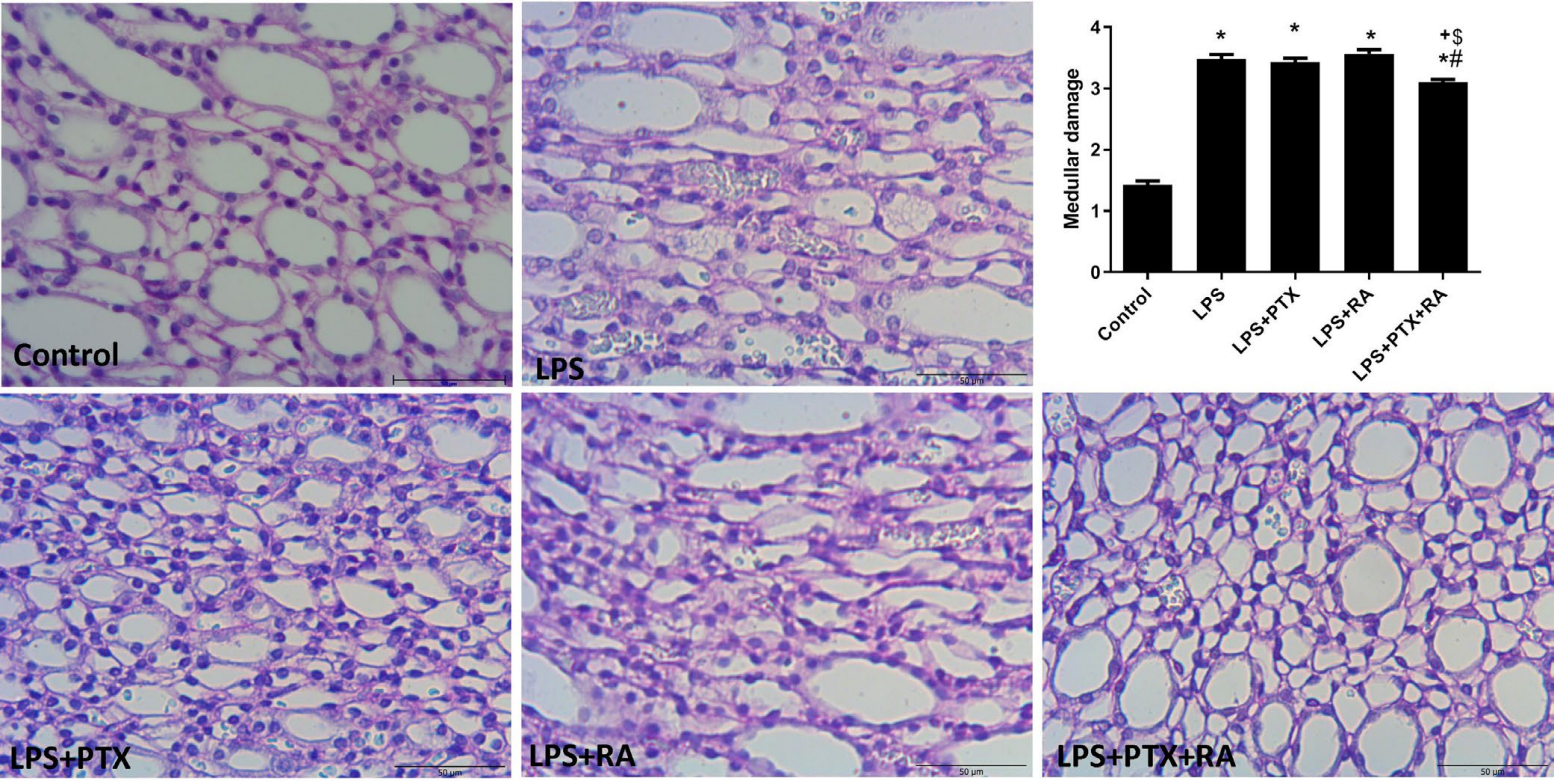
Representative images of renal medullary damage and damage score among the experimental groups. Values are represented as mean ± SEM. *p < 0.05 vs. Control, ^+^ *p* < 0.05 vs. LPS, ^#^ *p* < 0.05 vs. LPS + PTX, ^$^ *p* < 0.05 vs. LPS + RA.

**FIGURE 5:**
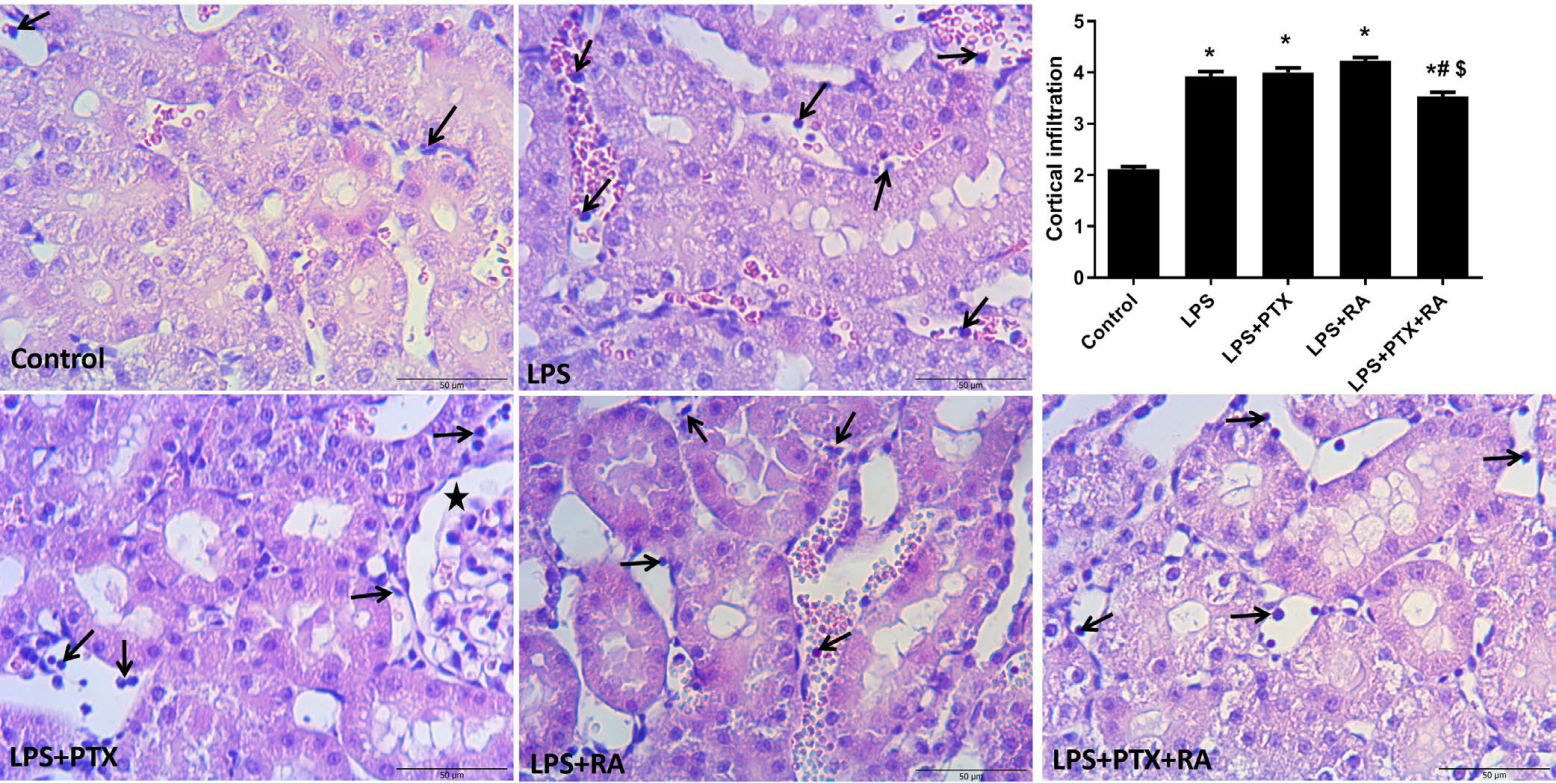
Representative images of cortical inflammatory cell infiltration and infiltration score among the experimental groups. Values are represented as mean ± SEM. *p < 0.05 vs. Control, ^#^ *p* < 0.05 vs. LPS+PTX, ^$^ *p* < 0.05 vs. LPS + RA.

**FIGURE 6:**
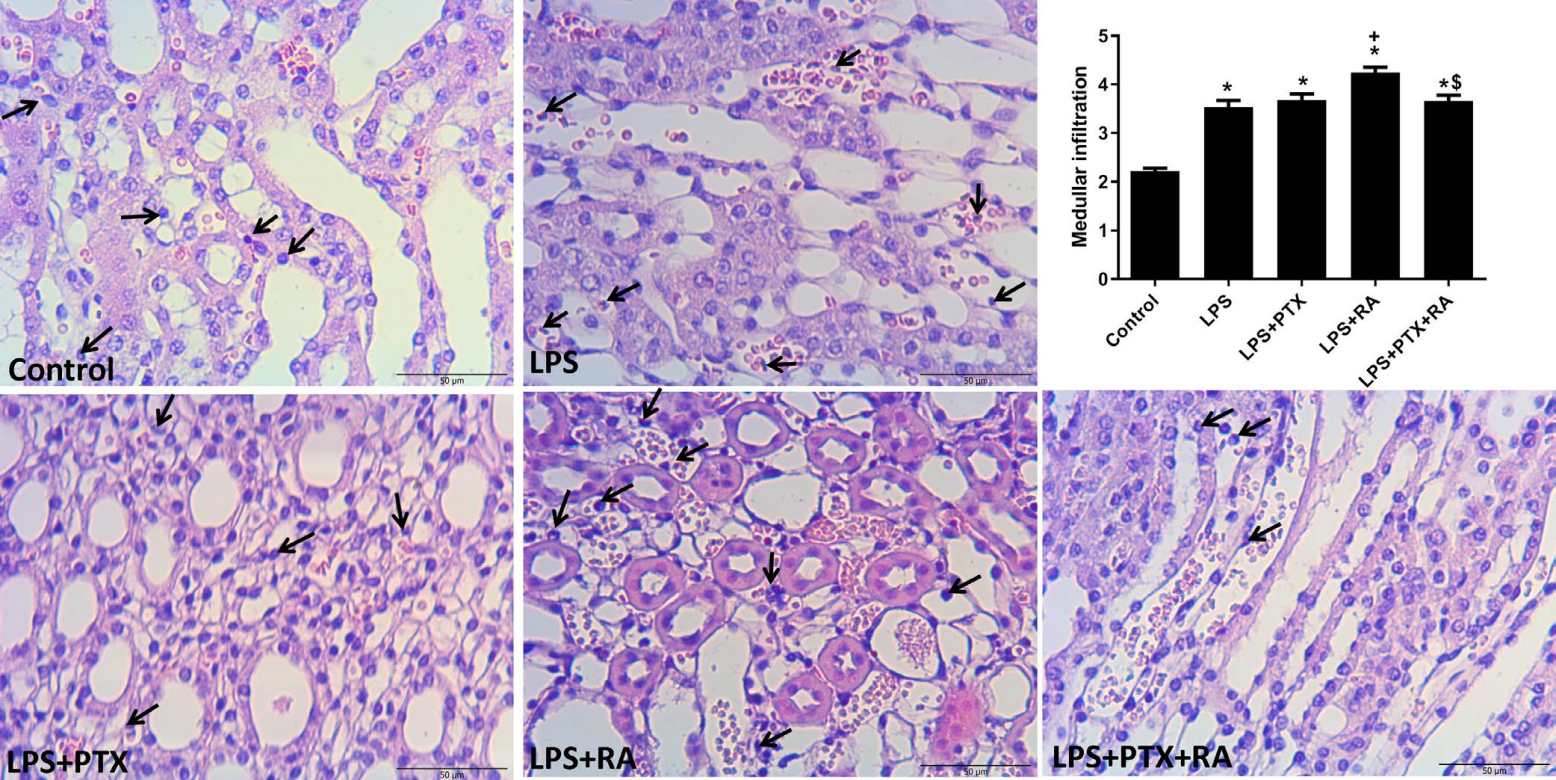
Representative images of medullary inflammatory cell infiltration and infiltration score among the experimental groups. Values are represented as mean ± SEM. *p < 0.05 vs. Control, ^+^ *p* < 0.05 vs. LPS, ^$^ *p* < 0.05 vs. LPS + RA.

Renal cortical damage was markedly increased in the LPS group in comparison to the Control (*p* < 0.05). However, treatment with PTX, RA, and PTX + RA led to decreased cortical damage with respect to the LPS group (*p* < 0.05) (Figure 3). Medullary damage was higher in all LPS groups than in the Control, but the damage was significantly reduced by the treatment of PTX + RA compared with the LPS, LPS + RA, and LPS + PTX groups (*p* < 0.05) (Figure 4). The renal cortical and medullary inflammatory cell infiltration was improved by using PTX and RA together compared to RA alone (*p* < 0.05) (Figures 5 and 6). The renal injury marker, NGAL, was significantly increased in the LPS group and the LPS groups that received RA or PTX with respect to the Control (*p* < 0.05) (Figure 8E), but not in the LPS group with combined treatment.

### 3.4 Muscle Microcirculation

**FIGURE 7:**
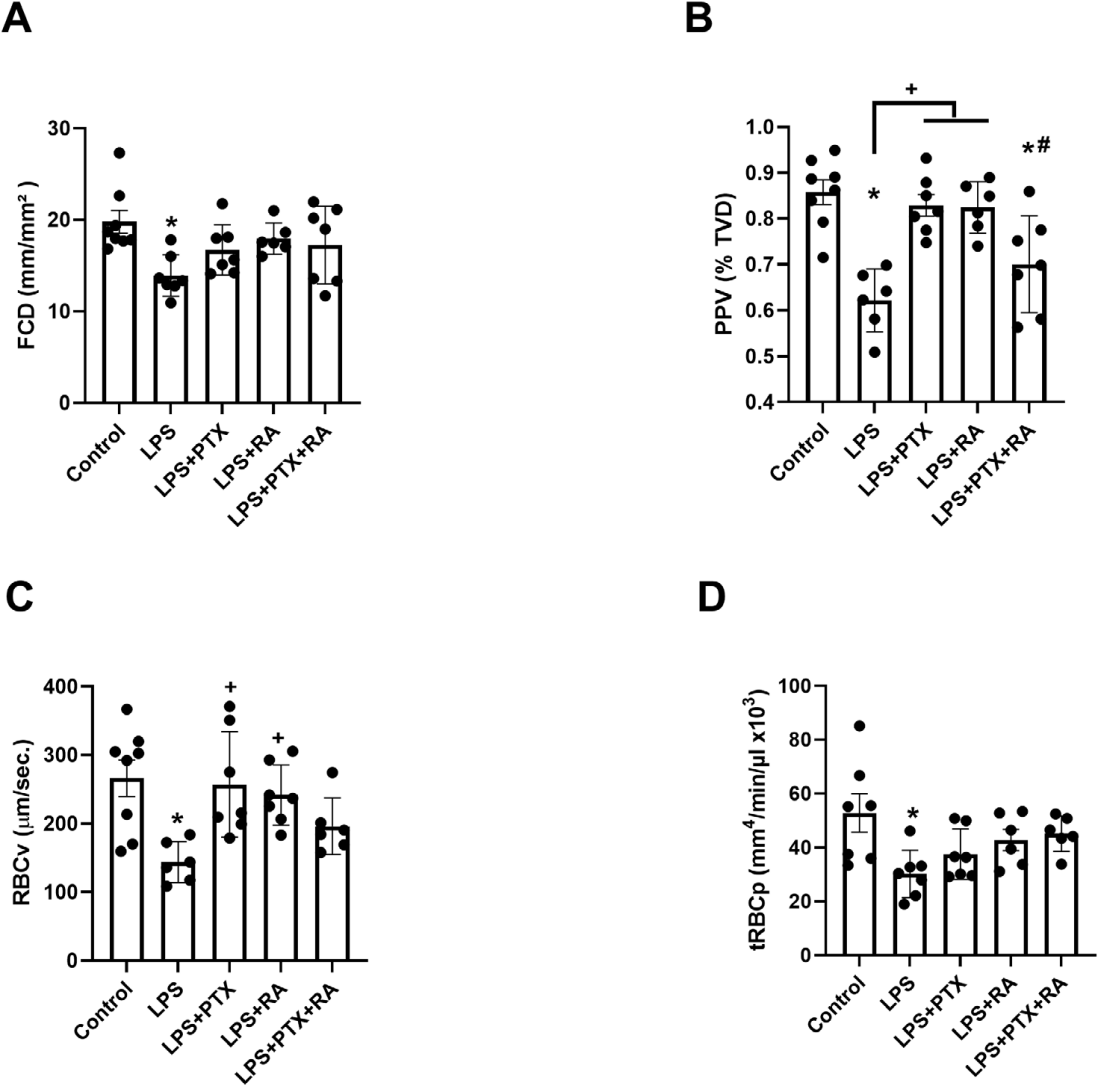
The muscle microcirculatory parameters. Functional capillary density (FCD) (A), proportion of perfused vessel (PPV) (B), red blood cell velocity (RBCv) (C), and tissue red blood cell perfusion (tRBCp) (D) at T4. Values are represented as mean ± SEM. *p < 0.05 vs. Control, ^+^ *p* < 0.05 vs. LPS, ^#^ *p* < 0.05 vs. LPS + PTX.

Despite FCD and tRBCp being decreased by LPS induction (*p* < 0.05 vs. Control), no significant alteration was found in the LPS groups that received PTX and RA at T4 compared to the control (Figure 7A,D). However, a decreased PPV and RBCv were significantly restored by PTX and RA therapies compared to the LPS group at T4 (*p* < 0.05) (Figure 7B,C).

### 3.5 Systemic Inflammation

**FIGURE 8:**
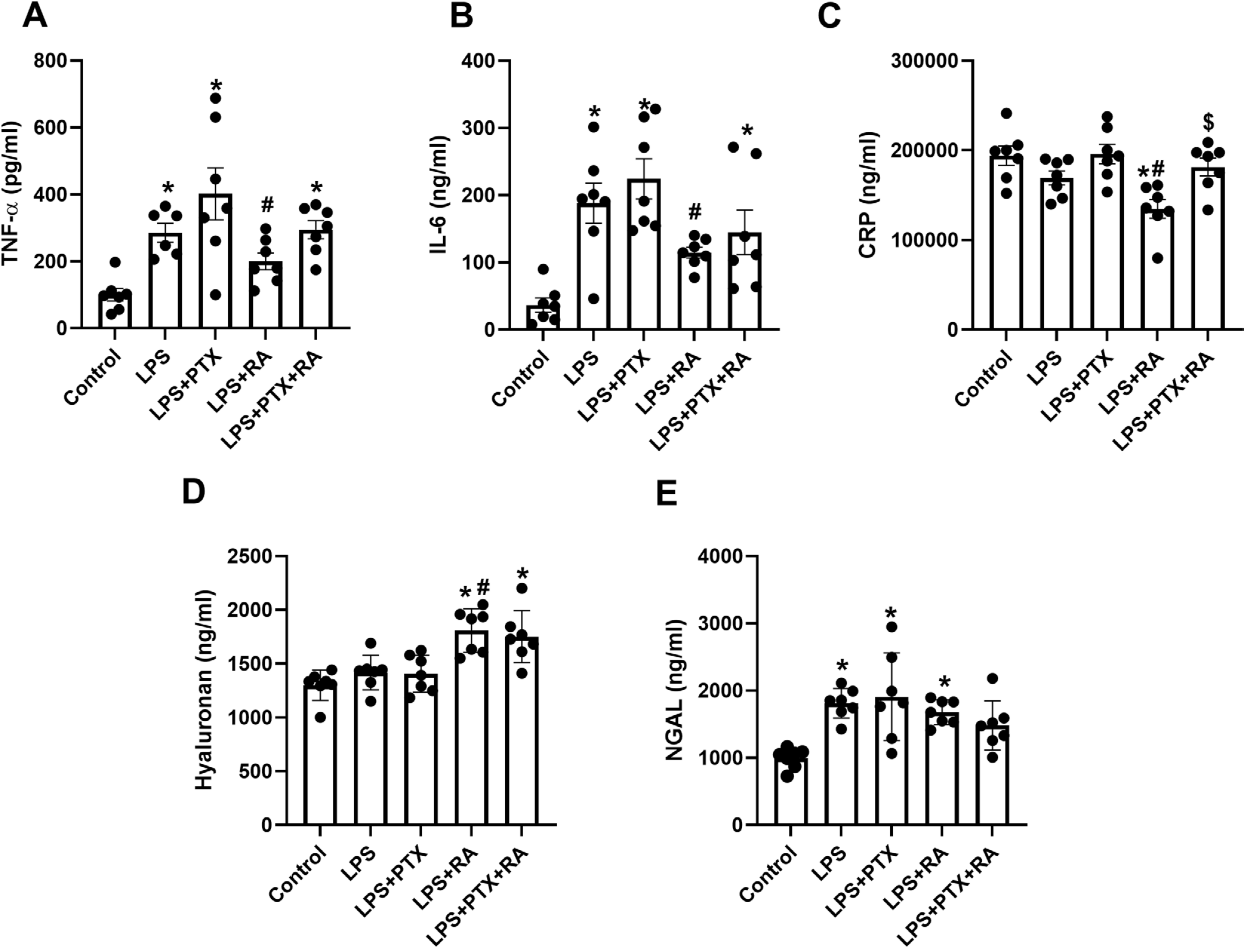
Plasma pro‐inflammatory markers. Tumor necrosis factor—alpha (TNF‐α) (A), interleukin‐6–6 (IL‐6) (B), and C‐reactive protein (CRP) (C); glycocalyx degradation marker, hyaluronan (D), plasma renal injury markers, neutrophil gelatinase‐associated lipocalin (NGAL) (E) at the end of experiments. Values are represented as mean ± SEM. *p < 0.05 vs. Control, ^+^ *p* < 0.05 vs. LPS, ^#^ *p* < 0.05 vs. LPS + PTX, ^$^ *p* < 0.05 vs. LPS + RA.

TNF‐α and IL‐6 levels were significantly increased in the LPS group, and the LPS group received both RA and PTX compared with the control (*p* < 0.05). However, TNF‐α level was higher in the LPS + PTX group than in the LPS + RA group (*p* < 0.05). Similarly, IL‐6 levels did not improve after PTX treatment; IL‐6 levels were lower in the LPS + RA group than in the PTX group (*p* < 0.05) (Figure 8A,B). However, CRP levels were improved only in the LPS group that received RA alone (*p* < 0.05 vs. LPS and LPS + PTX) (Figure 8C).

As a marker of glycocalyx degradation, plasma hyaluronan levels increased in LPS groups receiving RA and PTX + RA compared with the control (RA). Still, treatment with PTX improved the hyaluronan level compared to LPS + RA (*p* < 0.05) (Figure 8D).

## 4 Discussion

In this translational study, we tested the potential benefits of PTX on systemic hemodynamics, renal perfusion and oxygenation, peripheral (muscle) microcirculation, and the vascular endothelium in LPS‐induced sepsis and resuscitation. We found that PTX leads to increased HR and lactate levels from the perspective of systemic hemodynamics and oxygenation. Although PTX did not have a significant impact on RBF or rstO_2_, cμPO_2_ was the only tissue oxygenation parameter that improved following PTX therapy. The improvement of RBF, rstO_2_, and DO_2ren_ is likely related to fluid resuscitation. The parameters of microcirculatory diffusion (PPV and FCD), convection (RBCv), and perfusion (tRBCp) were protected by the PTX. Renal inflammatory cell infiltration was also decreased by the PTX combined with fluid resuscitation in both the cortex and medulla. However, using the PTX did not improve TNF‐α and IL‐6 levels when compared to fluid resuscitation alone.

In this study, although we did not find any adverse effect of PTX on arterial pressure, HR was considerably increased by PTX treatment. This positive chronotropic effect of PTX may be linked to increased cyclic adenosine monophosphate (cAMP) levels in cardiac cells [16]. A randomized placebo‐controlled study has shown that the use of PTX in patients with dilated cardiomyopathy is associated with an increase in left ventricular ejection fraction [17]. In another randomized, double‐blind, placebo‐controlled study, Staubach et al. also noted that using PTX, without adverse effects, improves pressure‐adjusted HR in patients with severe sepsis [18]. In an experimental traumatic hemorrhagic shock study, using 50 mg·kg^−1^ of PTX exerted the restoration of cardiac output and tissue perfusion [19]. However, the plasma lactate level, as a marker of tissue hypoxia, was unexpectedly increased by PTX in this study. This can be explained by restored tissue perfusion and the high discharge of lactate, which may be trapped in ischemic tissue after septic insult. In opposition to our findings, it is reported that PTX can improve lactate levels in a model of intra‐abdominal sepsis in rats [20]. Despite no data regarding peripheral organs, it might be considered that high cAMP levels may also induce lactate production by glycogen breakdown [9].

From the perspective of the effects of PTX on the kidney, Wang et al. demonstrated that PTX can enhance the renal glomerular filtration rate (GFR), reduce inflammation, and improve nitric oxide metabolism in mice [21]. The renal microcirculatory oxygenation and perfusion assessment of this study revealed that PTX can improve renal oxygen availability in the cortex but has no significant effect on RBF, DO_2ren_, and VO_2ren_. The effects of PTX on cortical oxygenation may be explained by prolonged nitric oxide activity resulting from phosphodiesterase inhibition [21, 22]. Of note, Zhang et al. showed that the additional PTX to fluid therapy can improve the PaO_2_, global DO_2_, and oxygen extraction in endotoxic dogs [23]. However, the present study shows that PTX has no prominent effects on renal blood flow, renal oxygen delivery, and consumption, as opposed to global oxygenation. The improvement of cμPO_2_ without prominent effects on RBF and DO_2ren_ following PTX treatment might be explained by the prevention of oxygen shunting from the cortex to the medullary region of the kidney. Indeed, Ellsworth et al. previously documented that tissue oxygen shunting may play a role in the redirection of oxygen from arterioles to venules [24]. In another experimental study, Legrand et al. also showed that renal ischemia/reperfusion is associated with increased medullary PO_2_ but decreased cortical PO_2_ during reperfusion, suggesting oxygen shunting [25]. Also, similar to our study, Gullichsen et al. demonstrated that experimental endotoxin infusion induced oxygen shunting, as evidenced by increased renal venous PO_2_ and decreased arteriovenous oxygen difference [26]. In addition to improving renal oxygen availability with PTX, the combination of PTX and fluid resuscitation reduced renal damage and renal inflammatory cell infiltration, as also reflected by plasma NGAL levels. Of note, the beneficial effects of PTX on sepsis‐induced microcirculatory perfusion and oxygenation defects might be augmented by using it in combination with the vasoconstrictors and antibiotics in clinical settings.

Concerning the results of muscle microcirculation, it has also been found that sepsis is associated with reduced microcirculatory diffusion (FCD and PPV), convection (RBCv), and perfusion (tRBCp) in rat muscle [4]. Following PTX injection, PPV was improved, and FCD, RBCv, and tRBCp were partly protected. These results underline the positive effect of PTX on peripheral microcirculation, facilitating tissue perfusion in long‐term use. Indeed, it is well documented that phosphodiesterase inhibition can promote vasodilation in the microcirculation by increasing cyclic guanosine monophosphate (cGMP) [21] and prolonging nitric oxide signaling [22] in a rat model of sepsis. This also explains the beneficial effect of PTX on intermittent claudication despite the lack of high‐certainty evidence based on the meta‐analysis about the use of PTX on walking capacity [8, 27]. However, a recent meta‐analysis has shown that PTX increases the maximum and pain‐free walking distance with a low ratio of adverse events [28]. In neonatal sepsis, similar to a clinical study [29], Pammi and Haque demonstrated that adjuvant PTX therapy in neonatal sepsis resulted in low mortality and hospital stay, but with low certainty of evidence [30].

In parallel with our findings regarding the inflammatory effects of PTX, no significant change was found in hyaluronan, TNF‐α, and IL‐6 levels between patients with non‐alcoholic steatohepatitis treated with PTX or placebo [31]. Moreover, serum endotoxin levels, TNF‐α, and IL‐6 levels were not different between the PTX and placebo groups in patients with severe sepsis [18]. In contrast, Speer et al. demonstrated that PTX combined with the antibiotics could improve both systemic‐renal inflammation, including TNF and IL‐6, and renal injury (NGAL and KIM‐1) in a neonatal murine model of *E. coli* sepsis [32]. Furthermore, despite PTX being considered a TNF inhibitor [20, 33], this effect is not observed in this experimental study. Conflicting data regarding the functional and inflammatory effects of PTX require an extensive mechanistic approach in future research.

In conclusion, we found that PTX may be protective of systemic hemodynamics, renal oxygenation, peripheral (muscle) microcirculation, renal damage, and renal inflammatory cell infiltration when it is combined with fluid resuscitation; however, it does not affect inflammation or renal function in the short term during LPS‐induced sepsis. Further experimental and clinical studies are needed to investigate the mechanism of action of PTX and confirm its long‐term effects on sepsis‐induced organ and peripheral microcirculatory dysfunction.

### 4.1 Perspectives

Sepsis is associated with hypotension, tissue/organ hypoperfusion, and microcirculatory dysfunction. Despite successful hemodynamic management, the microcirculatory dysfunction persists and leads to multiple organ failure in the ICU. Pentoxifylline, as a non‐selective phosphodiesterase inhibitor, may attenuate deterioration in peripheral/renal microvascular perfusion and oxygenation. This study demonstrates that Pentoxifylline exhibits a protective role on the peripheral perfusion, renal microcirculatory oxygenation, and renal damage.

## Funding

The study is supported by the internal department of Erasmus MC.

## Ethics Statement

This study was approved by the National Committee of Animal Experimentation (CCD172326, approval date: 05‐03‐2018) and the Animal Research Committee of the Erasmus Medical Centre, Rotterdam (SP2100292).

## Conflicts of Interest

The authors declare no conflicts of interest.

## Data Availability

Data sharing not applicable to this article as no datasets were generated or analyzed during the current study.
